# Attention–deficit/hyperactivity disorder symptoms, perceived stress, and suicidal ideation during the COVID-19 pandemic

**DOI:** 10.3389/fpsyt.2022.1008290

**Published:** 2022-11-09

**Authors:** Andrew Stickley, Aya Shirama, Takuma Inagawa, Vladislav Ruchkin, Roman Koposov, Johan Isaksson, Yosuke Inoue, Tomiki Sumiyoshi

**Affiliations:** ^1^Department of Preventive Intervention for Psychiatric Disorders, National Center of Neurology and Psychiatry, Tokyo, Japan; ^2^Department of Psychiatry, National Center Hospital, National Center of Neurology and Psychiatry, Tokyo, Japan; ^3^Department of Medical Sciences, Unit of Child and Adolescent Psychiatry, Uppsala University, Uppsala, Sweden; ^4^Sala Forensic Psychiatric Clinic, Sala, Sweden; ^5^Regional Center for Child and Youth Mental Health and Child Welfare, Faculty of Health Sciences, UiT the Arctic University of Norway, Tromsø, Norway; ^6^M. Sechenov First Moscow State Medical University, Moscow, Russia; ^7^Karolinska Institutet Center of Neurodevelopmental Disorders (KIND), Center for Psychiatry Research, Department of Women's and Children's Health, Karolinska Institutet and Stockholm Health Care Services, Region Stockholm, Stockholm, Sweden; ^8^Department of Epidemiology and Prevention, Center for Clinical Sciences, National Center for Global Health and Medicine, Tokyo, Japan

**Keywords:** ADHD, COVID-19, mental health, self–perceived stress, suicide

## Abstract

**Objective:**

Attention–deficit/hyperactivity disorder (ADHD) symptoms have been linked to suicidal behavior. However, little is known about the mechanisms involved in this association. This study examined ADHD symptoms and suicidal ideation during the COVID-19 pandemic and the role of self–perceived stress in this association.

**Method:**

Data were analyzed from an online sample of 1,452 Japanese individuals aged 18 to 89 obtained in February 2021. Information was collected on past–year suicidal ideation. ADHD symptoms were assessed with the Adult ADHD Self–Report Scale (ASRS) Screener while stress was measured with the Perceived Stress Scale (PSS−14). Depressive symptoms were assessed with the Patient Health Questionnaire (PHQ−9), while the Generalized Anxiety Disorder scale (GAD−7) was used to assess anxiety symptoms. Logistic regression was used to examine associations.

**Results:**

Fifty–one (3.5%) individuals had ADHD symptoms, while more than one in ten (11.7%) of the respondents reported past–year suicidal ideation. In an analysis adjusted for sociodemographic factors, ADHD symptoms were associated with eight times higher odds for past–year suicidal ideation. However, adjusting the analysis for mental health variables (anxiety and depressive symptoms) attenuated the association which became non–significant (odds ratio [OR]: 1.96, 95% confidence interval [CI]: 0.92–4.18). In contrast, in an analysis adjusted for mental health, individuals with ADHD symptoms and stress had significantly higher odds for suicidal ideation (OR: 3.72, 95%CI: 1.43–9.67) whereas, ADHD symptoms without stress were not linked to suicidal ideation (OR: 1.25, 95%CI: 0.38–4.18).

**Conclusions:**

Self–perceived stress is important in the association between ADHD symptoms and suicidal ideation among individuals in Japan during the COVID-19 pandemic. Detecting and managing stress and its effects in individuals with ADHD/ADHD symptoms should be a therapeutic focus for improving health–related outcomes in this population both during the COVID-19 pandemic and beyond.

## Introduction

Attention–deficit/hyperactivity disorder (ADHD) is a childhood–onset neurodevelopmental disorder characterized by high and impairing levels of inattention and/or hyperactivity–impulsivity (1). It has been estimated that ADHD symptoms persist into adulthood in varying degrees in a majority of individuals (around 65%) (2), with 2.5–3.4% of adults having the disorder (3). A growing body of research has shown that ADHD is associated with detrimental outcomes across a range of spheres in adulthood. In particular, adults with ADHD are more likely to encounter problems associated with work (e.g., non–employment, lower job status, increased risk of dismissal) (4, 5) and family life (e.g., have poorer quality marriages that are more likely to end in divorce) (6, 7). Adult ADHD has also been linked to poorer physical and mental health (8, 9). Regarding the latter, a recent study using data from 20 nationally/regionally representative WHO World Mental Health Surveys found that adults with ADHD were more likely to have comorbid mood, anxiety, behavior and substance use disorders (10).

There is also some evidence that adult ADHD may be linked to an increased risk for suicidal behavior. An early review study reported that ADHD appeared to increase the risk of suicide but that it was largely through its detrimental effects on comorbid conditions such as depression (11). Subsequent reviews have also found an association between ADHD and suicide and highlighted the role of comorbid disorders in this association to a greater or lesser degree (12–14), while a recent systematic review and meta–analysis found an association between ADHD and suicidal ideation and suicide attempts even after pooling odds ratios that had been adjusted for a range of variables (15). Other recent research has further extended understanding of the association between ADHD and suicide. For example, a study using nationwide register data for the whole of the Danish population aged ≥10 found that although individuals with an ADHD diagnosis had a four–fold higher rate of suicidal behavior, among those with ADHD and any comorbid psychiatric disorders the rate was substantially higher (16). In addition, research has further indicated that psychological constructs such as self–esteem might also be important for the association between ADHD and suicidality (17).

Building on earlier research, which has shown that ADHD symptoms are associated with suicidal behavior in both general and clinical populations (18, 19), this study will examine the association between ADHD symptoms and suicidal ideation in Japanese community–based adults during the COVID-19 pandemic and the role of self–perceived stress in this association. This research may be timely for several reasons. Previous studies have found that ADHD symptoms are linked to higher levels of perceived stress in adults (20, 21) and that perceived stress may play a role in poor mental health outcomes in adults with ADHD symptoms (22). Importantly, the coronavirus pandemic has been associated with the occurrence of high levels of stress across numerous life spheres (23), including for many people in Japan (24). It has also been linked to a deterioration in mental health with research showing an increase in depressive, anxiety and insomnia symptoms (25), which may be related to COVID-19 preventive policies such as lockdowns (26) as well as fear of COVID-19, with a recent meta–analysis linking COVID-19–related fear to anxiety, depression, distress, stress and traumatic stress (27). In addition, biological factors might also be important in this context as there is some evidence that inflammation associated with the immune response to COVID-19 might also be linked to worse mental health (28), with the upregulation of specific cytokines (TNF–α and IL−6) possibly resulting in mood changes and non–functional stress–related responses (29). Moreover, there is also some evidence that people with more ADHD symptoms may have been more likely to experience psychological distress during the COVID-19 pandemic (30). This is worrying given that worse mental health has also been associated with suicidal ideation in Japan during this period (31). In short, although there has been comparatively little research as yet that has focused specifically on adults with ADHD during the COVID-19 pandemic, it is possible that Japanese individuals with ADHD symptoms may have an increased risk for both stress and suicidal ideation during this period.

The two previous studies that have examined the association between ADHD symptoms, stress and suicidal ideation both found that perceived stress partially accounted for the association between ADHD symptoms and suicidal ideation. However, both studies focused on subpopulations—students (32) and emerging adults (33) in the pre–pandemic period. Thus, the role of stress in the association between ADHD symptoms and suicidal ideation among all–age adults remains uncertain, especially in the context of a potentially highly stressful environment. Given this, the current study had three main aims. To: (i) examine if ADHD symptoms are associated with stress among Japanese individuals during the COVID-19 pandemic; (ii) determine if ADHD symptoms are linked to suicidal ideation during this period; and (iii) explore whether perceived stress is important for the association between ADHD symptoms and suicidal ideation.

## Methods

### Study sample

Data were analyzed from an online survey of the Japanese general population that was undertaken over a 2–day period in late February, 2021. At the time there was a state of emergency in place in 10 of Japan's 47 prefectures, with companies being encouraged to allow working from home, restaurants and bars being asked to shut by 8 pm and people told to stay at home whenever possible (34, 35). The survey was administered by Macromill, a commercial survey company. A questionnaire was initially sent to 8,628 respondents and then to an additional 1,728 respondents that comprised part of the company's online commercial web panel. A 16–cell monitoring selection procedure was used to construct a sample of 1,452 respondents based on several inclusion criteria i.e., that respondents were aged 18 or above, that the sex distribution was representative of the broader Japanese population and that respondents were drawn from all of Japan's prefectures. There were no specific exclusion criteria as it was assumed that the online sample would not include individuals who are often excluded from surveys such as those who are institutionalized, hospitalized, incarcerated, or homeless. Approval for the survey was obtained from the Ethics Committee at the National Center of Neurology and Psychiatry, Tokyo, Japan (approval number: A2020–088). Informed consent was obtained from all participants.

### Measures

#### Suicidal ideation

Suicidal ideation was assessed with a two–part question. Respondents were initially asked, “Have you ever thought of taking your life, even if you would not really do it?” with yes and no answer options. Those who responded in the affirmative were then asked a follow–up question, “Was this… (i) in the last year (scored 1); (ii) or at some other time (scored 2)”. Those who scored 1 on this follow–up question were categorized as having past–year suicidal ideation. This question is modeled on an earlier question that was used when examining the association between ADHD symptoms and suicidal behavior in the general population (18).

#### ADHD symptoms

ADHD symptoms were assessed with the Adult ADHD Self–Report Scale (ASRS) Screener (36, 37). This 6–item screening scale uses a 5–point response option that runs from never (scored 0) to very often (scored 4) to enquire about inattention and hyperactivity in the previous 6 months. The total score can range from 0 to 24 with higher scores indicating increased ADHD symptoms. Following the lead of the scale's developers, in the current study a score of 14 and above was used to categorize ADHD symptoms (37). Cronbach's alpha for the scale was 0.87.

#### Stress

The level of respondents' stress was assessed with the Perceived Stress Scale (PSS−14) (38). This 14–item self–report scale is used to assess the degree to which past–month situations in an individual's life are appraised as stressful. The scale consists of seven negative and seven positive (reverse scored) items with response options ranging from “never” (scored 0) to very often (scored 4). The total scale score ranges from 0 to 56 with higher scores indicating increased levels of stress. Previous research has reported that the scale's psychometric properties are acceptable (39) and that the scale is valid for use in Japan (40). In order to focus on those individuals with the highest levels of stress, in the current study the top decile of scores was chosen as a cut–off point (a score of 37 and above) to categorize experiencing stress. Cronbach's alpha for the scale was 0.86.

#### Covariates

Information was also obtained on several other variables. Besides sex (male, female) respondents were categorized into three age groups, 18–34, 35–59 and 60 and above. Educational level was dichotomized into higher education (2–year college, university, graduate school) and less than higher education (junior high school/below, high school, vocational high school). Marital status was also classified using two categories: married or not married. Household financial income was measured in millions of yen and assessed using three categories, (i) <4 million, (ii) 4 < 10 million, and (iii) ≥10 million (106.55 JPY = U.S. $1 at the time of the survey). Given the large number of respondents that refused to answer this question (22.7%), and our desire to keep as many people in the analysis as possible, a fourth (iv) “missing” category was also created. Depressive symptoms in the past 2 weeks were assessed with the self–report Patient Health Questionnaire (PHQ−9) (41). The score of this 9–item scale can range between 0 and 27 with higher scores indicating increased depressive symptoms. As the ninth item of the PHQ−9 assesses suicidal ideation, in the current study this item was removed and the 8–item version of the scale (PHQ−8) was used. A cut–off score of 10 and above was used to categorize depression following the lead of an earlier study which determined that this cut–off was appropriate for assessing current depression when using the PHQ−8 in population–based studies (42). The self–report Generalized Anxiety Disorder scale (GAD−7) was used to assess anxiety symptoms in the past 2 weeks (43). This 7–item scale produces a total score that can range between 0 and 21 with higher scores indicating increased anxiety symptoms. Following the recommendation of the scale's developers a score of 10 and above was used in the current study to categorize at least a moderate level of anxiety (43).

### Statistical analysis

Descriptive statistics of the study sample stratified by ADHD symptoms status were first calculated. Next, logistic regression analysis was used to examine the association between ADHD symptoms and self–perceived stress. Three models were used in the analysis. In Model 1 the bivariate association between ADHD symptoms and stress was examined. Model 2 included sociodemographic (sex, age, education, marital status, family household income) variables. The fully adjusted Model 3 included the same variables as Model 2 and mental health variables (depressive and anxiety symptoms). Logistic regression analysis was then used to examine the association between ADHD symptoms and suicidal ideation in the past year using the same three–model building process previously described. Finally, logistic regression analysis was again used to examine the associations between ADHD symptoms, suicidal ideation and stress in the context of mental health. To do this a four–category ADHD symptoms–stress variable was created. The reference category included individuals without ADHD symptoms or stress. Category two included individuals experiencing stress only, while category three included individuals with ADHD symptoms only. The fourth category comprised those individuals with both ADHD symptoms and stress. The same analytic model building process was used as in the previous analyses. All analyses were performed with the Statistical Package for the Social Sciences (SPSS) version 24 and were adjusted for location (prefecture). The results are presented as odds ratios (OR) with 95% confidence intervals (CI). The level of statistical significance was set as *p* < 0.05 (two–tailed).

## Results

The analytic sample comprised 1,452 respondents with an average age of 51.6 years (range 18 to 89), with slightly more than half of them being female (51.5%) (Table 1). A majority of the respondents were married (61.3%), had received a higher education (64.5%) and had a family income in the four to 10 million yen range (41.3%). When using the 14 and above cut–off point, 3.5% (*N* = 51) of the sample was categorized as having ADHD symptoms. Mental health conditions were prevalent in the sample with 15.0% of the respondents categorized as having depressive symptoms, while the corresponding figure for anxiety symptoms was 10.8%. Just over one in ten participants (11.7%) reported past–year suicidal ideation. Chi-square tests showed that ADHD symptoms were associated with younger age, not being married, anxiety and depressive symptoms, stress symptoms and reporting past–year suicidal ideation.

**Table 1:** Sample characteristics by ADHD status.

**Variable**	**Total**	**No ADHD symptoms**	**ADHD symptoms**	***P*** **–value**
	***N*** **(%)**	***N*** **(%)**	***N*** **(%)**	
Sex				0.622
Male	704 (48.5)	681 (48.6)	23 (45.1)	
Female	748 (51.5)	720 (51.4)	28 (54.9)	
Age				<0.001
18–34	322 (22.2)	293 (20.9)	29 (56.9)	
35–59	572 (39.4)	557 (39.8)	15 (29.4)	
≥60	558 (38.4)	551(39.3)	7 (13.7)	
Education				0.534
Higher education	937 (64.5)	902 (64.4)	35 (68.6)	
<Higher education	515 (35.5)	499 (35.6)	16 (31.4)	
Marital status				<0.001
Married	890 (61.3)	872 (62.2)	18 (35.3)	
Not married	562 (38.7)	529 (37.8)	33 (64.7)	
Household income (Yen)				0.293
<4 million	415 (28.6)	399 (28.5)	16 (31.4)	
4 million to <10 million	599 (41.3)	584 (41.7)	15 (29.4)	
≥10 million	109 (7.5)	105 (7.5)	4 (7.8)	
Missing data	329 (22.7)	313 (22.3)	16 (31.4)	
Depressive symptoms				<0.001
No	1234 (85.0)	1224 (87.4)	10 (19.6)	
Yes	218 (15.0)	177 (12.6)	41 (80.4)	
Anxiety symptoms				<0.001
No	1295 (89.2)	1278 (91.2)	17 (33.3)	
Yes	157 (10.8)	123 (8.8)	34 (66.7)	
Stress symptoms				<0.001
No	1307 (90.0)	1287 (91.9)	20 (39.2)	
Yes	145 (10.0)	114 (8.1)	31 (60.8)	
Past–year suicidal ideation				<0.001
No	1282 (88.3)	1257 (89.7)	25 (49.0)	
Yes	170 (11.7)	144 (10.3)	26 (51.0)	

ADHD, attention–deficit/hyperactivity disorder.

In a bivariate analysis individuals with ADHD symptoms had over twenty times higher odds for experiencing stress (OR: 24.22, 95%CI: 12.06–48.64) (Model 1, Table 2). Including sociodemographic variables in the analysis attenuated this association slightly (Model 2). The further inclusion of mental health variables in Model 3 led to a large reduction in the ADHD OR. Nonetheless, individuals with ADHD symptoms still had over five times higher odds for experiencing stress compared to individuals without ADHD symptoms. Anxiety and depressive symptoms were both associated with significantly higher odds for experiencing stress with the OR for the latter being similar to that for ADHD symptoms (6.09 vs. 5.19).

**Table 2:** Association between ADHD symptoms and perceived stress in Japan during the COVID-19 pandemic (*N* = 1,452).

	**Model 1**	**Model 2**	**Model 3**
	**OR (95%CI)**	**OR (95%CI)**	**OR (95%CI)**
ADHD symptoms	24.22 (12.06–48.64)^***^	21.77 (10.56–44.88)^***^	5.19 (2.27–11.84)^***^
Sex (Female)		1.24 (0.83–1.84)	1.29 (0.84–1.98)
Age			
18–34		Ref.	Ref.
35–59		1.30 (0.80–2.10)	1.79 (1.04–3.07)^*^
≥60		0.35 (0.19–0.64)^**^	0.61 (0.31–1.20)
Education			
<Higher education		1.09 (0.72–1.64)	1.06 (0.67–1.67)
Marital status			
Not married		1.56 (1.01–2.41)^*^	1.35 (0.84–2.18)
Household income (Yen)			
≥10 million		Ref.	Ref.
<4 million		1.79 (0.70–4.62)	1.82 (0.64–5.22)
4 million to <10 million		1.76 (0.72–4.31)	2.55 (0.94–6.96)
Missing data		1.45 (0.56–3.73)	2.03 (0.71–5.81)
Depression			6.09 (3.60–10.30)^***^
Anxiety			3.46 (1.97–6.07)^***^

ADHD, Attention–deficit/hyperactivity disorder.

OR, Odds ratio; CI, Confidence interval.

Ref, Reference category.

*** p < 0.001,

** p < 0.01,

* p < 0.05.

All models were adjusted for prefecture.

Individuals with ADHD symptoms had over 10 times higher odds for past–year suicidal ideation in a bivariate analysis (OR: 10.78, 95%CI: 5.69–20.43) (Model 1, Table 3). The inclusion of sociodemographic variables attenuated this association although individuals with ADHD symptoms continued to have over eight times higher odds for suicidal ideation (Model 2). However, the inclusion of the anxiety and depressive symptoms variables in Model 3 resulted in the association between ADHD symptoms and suicidal ideation becoming non–significant (OR: 1.96, 95%CI: 0.92–4.18). This reduction in odds seems to have been principally due to the depressive symptoms variable, which was associated with over eight times higher odds for suicidal ideation.

**Table 3:** Association between ADHD symptoms and suicidal ideation in Japan during the COVID-19 pandemic (*N* = 1,452).

	**Model 1**	**Model 2**	**Model 3**
	**OR (95%CI)**	**OR (95%CI)**	**OR (95%CI)**
ADHD symptoms	10.78 (5.69–20.43)^***^	8.08 (4.16–15.71)^***^	1.96 (0.92–4.18)
Sex (Female)		1.43 (1.00–2.06)	1.50 (1.01–2.22)^*^
Age			
18–34		Ref.	Ref.
35–59		0.67 (0.44–1.03)	0.77 (0.49–1.23)
≥60		0.18 (0.11–0.32)^***^	0.28 (0.15–0.51)^***^
Education			
<Higher education		1.09 (0.74–1.60)	1.08 (0.71–1.64)
Marital status			
Not married		1.31 (0.88–1.94)	1.04 (0.67–1.60)
Household income (Yen)			
≥10 million		Ref.	Ref.
<4 million		1.41 (0.65–3.06)	1.35 (0.58–3.13)
4 million to <10 million		0.89 (0.43–1.88)	1.05 (0.47–2.34)
Missing data		0.82 (0.38–1.80)	0.95 (0.41–2.21)
Depression			8.15 (5.01–13.27)^***^
Anxiety			1.58 (0.92–2.71)

ADHD, Attention–deficit/hyperactivity disorder.

OR, Odds ratio; CI, Confidence interval.

Ref, Reference category.

*** p < 0.001,

* p < 0.05.

All models were adjusted for prefecture.

Compared to individuals with no ADHD symptoms and no stress, individuals with stress only and ADHD symptoms only both had over five times higher odds for suicidal ideation in a bivariate analysis (Model 1, Table 4), while those respondents with both ADHD symptoms and stress had over 20 times higher odds for suicidal ideation (OR: 21.46, 95%CI: 9.57–48.13). The inclusion of sociodemographic variables reduced the odds slightly across all of these categories. The inclusion of mental health variables in Model 3 fully attenuated the association between ADHD symptoms only and suicidal ideation (OR: 1.25, 95%CI: 0.38–4.18). In contrast, individuals with stress only had almost two times higher odds for suicidal ideation (OR: 1.94, 95%CI: 1.13–3.35), while those with both ADHD symptoms and stress had over 3.7 times higher odds for suicidal ideation. The only other variables that were associated with suicidal ideation in the fully adjusted Model 3 were depressive symptoms (OR: 7.23, 95%CI: 4.38–11.93) and older age which was associated with a 72% reduction in the odds for suicidal ideation compared with the 18–34 reference category.

**Table 4:** Association between ADHD symptoms, perceived stress and suicidal ideation in Japan during the COVID-19 pandemic (*N* = 1,452).

	**Model 1**	**Model 2**	**Model 3**
	**OR (95%CI)**	**OR (95%CI)**	**OR (95%CI)**
ADHD			
No ADHD–No Stress	Ref.	Ref.	Ref.
Stress only	5.78 (3.66–9.12)^***^	4.68 (2.91–7.53)^***^	1.94 (1.13–3.35)^*^
ADHD symptoms only	6.54 (2.26–18.98)^**^	4.83 (1.59–14.63)^**^	1.25 (0.38–4.18)
ADHD symptoms+Stress	21.46 (9.57–48.13)^***^	15.39 (6.67–35.52)^***^	3.72 (1.43–9.67)^**^
Sex (Female)		1.38 (0.95–2.00)	1.45 (0.97–2.15)
Age			
18–34		Ref.	Ref.
35–59		0.62 (0.40–0.96)^*^	0.73 (0.46–1.17)
≥60		0.21 (0.12–0.37)^***^	0.28 (0.15–0.51)^***^
**Education**			
<Higher education		1.05 (0.71–1.57)	1.08 (0.71–1.65)
**Marital status**			
Not married		1.18 (0.78–1.78)	0.98 (0.63–1.53)
**Household income (Yen)**			
≥10 million		Ref.	Ref.
<4 million		1.26 (0.58–2.75)	1.30 (0.56–3.00)
4 million to <10 million		0.77 (0.37–1.63)	0.95 (0.42–2.11)
Missing data		0.73 (0.33–1.62)	0.86 (0.37–2.00)
Depression			7.23 (4.38–11.93)^***^
Anxiety			1.36 (0.78–2.38)

ADHD, Attention–deficit/hyperactivity disorder.

OR, Odds ratio; CI, Confidence interval.

Ref, Reference category.

*** p < 0.001,

** p < 0.01,

* p < 0.05.

All models were adjusted for prefecture.

## Discussion

This study used data from an online survey of 1,452 Japanese individuals aged 18 and above to examine the link between ADHD symptoms and suicidal ideation and the role of perceived stress in this association during the COVID-19 pandemic. Results from logistic regression analyses showed that ADHD symptoms were strongly associated with both perceived stress and suicidal ideation although mental health (depressive symptoms) mediated the association between ADHD symptoms and suicidal ideation. Further analyses also showed that when controlling for mental health problems, individuals with comorbid ADHD symptoms and stress had significantly higher odds for suicidal ideation compared to those with no ADHD symptoms or stress, while ADHD symptoms with no stress were not associated with suicidal ideation in the fully adjusted analysis.

The finding that ADHD symptoms are associated with higher levels of perceived stress accords with the findings of previous research, which has shown that individuals with ADHD/ADHD symptoms may be especially vulnerable to experiencing stress (20, 21, 44). It has been speculated that higher levels of perceived stress may be related to the impairments that individuals with ADHD can experience across different life domains (20). This is supported by research which has shown that higher levels of self–perceived stress in ADHD are correlated with a greater number of stressors in everyday life (44) and that ADHD symptoms are associated with higher odds for a range of stressful life events including relationship, family and employment problems (45). Given that self–perceived stress is possibly elevated in many individuals with ADHD symptoms, it is difficult to determine whether this situation may have been further exacerbated during the COVID-19 pandemic—especially given the lack of COVID-19–related research on adults with ADHD/ADHD symptoms to date. Nonetheless, there is some indication that this might be the case. For example, a study from Israel from early in the pandemic linked ADHD symptom levels to experiencing financial problems (30), which can act as a source of stress in individuals with ADHD symptoms (45). A small qualitative study from Japan also found that the pandemic may have worsened the effects of adult ADHD symptoms (46) with the possibility this carries for negative outcomes and associated stress.

In an analysis adjusted for sociodemographic factors individuals with ADHD symptoms had over eight times higher odds for reporting suicidal ideation. However, when the analysis was adjusted for mental health problems the association became non–significant with depressive symptoms having an especially strong relationship with suicidal ideation. Earlier studies have produced conflicting results on the role of comorbid mental disorders in the association between ADHD symptoms and suicidality (18, 47). However, the results from the current study seem to support the finding that problems with mental health (depressive symptoms) may be an important mediator in the association between ADHD symptoms and suicidal ideation (48). It is unclear whether ADHD exacerbates the severity of depressive symptoms and in that way increases the risk for suicidal behavior (11) or whether other mechanisms such as deficits in emotion regulation (48) might be involved in this process. Regardless, the fact that ADHD symptoms are often comorbid with depressive symptoms/depression (49, 50) and that depression is associated with not only suicide ideation but also attempted and completed suicide (51), highlights the importance of both identifying ADHD in adults and treating comorbid disorders in adults with ADHD (52).

Individuals with ADHD symptoms and stress had over 3.5 times higher odds for suicidal ideation in a fully adjusted analysis. However, the inclusion of mental health problems in the analysis attenuated the association between ADHD symptoms only and suicidal ideation which became non–significant. This result concurs with earlier research which has shown that stress is associated with suicidal ideation (53) and that perceived stress may be important in the association between ADHD symptoms and suicidal ideation (32, 33). Indeed, it is possible that the detrimental effects of stress might be especially important at the present time given that COVID-19–related stress symptoms have been linked with suicidal ideation/self–harm in the general population (54). Various mechanisms might underlie the association between ADHD symptoms, stress and suicidal ideation. In particular, a recent study reported that a negative response to stress i.e., stress–reactive rumination might be important in this context (33). Alternatively, other factors might be involved. It can be speculated for example, that the impaired decision–making and problem–solving ability observed in adults with ADHD (13, 55) might also be relevant given that other research has shown that low problem solving ability might be important for the association between life stress and suicidal ideation (56). Given the findings of this study prospective research is now warranted to both confirm and further elucidate the role of stress in the association between ADHD symptoms and suicidal behavior.

This study has several limitations that should be mentioned. The study data came from an online sample which may be susceptible to selection bias due to processes such as self–selection and non–coverage (e.g., through not having access to the Internet) (57). Having said this, information from the 2020 Japanese census indicated that that proportion of females in our sample was almost identical to the population proportion (51.5–51.4%), the average age of our respondents was slightly older than the population average (51.6 > 47.6 years), the proportion married slightly exceeded the figure for the total Japanese population (61.3 > 55.6%) (58), while the average household income in 2018 (5.52 million yen) fell within the income category with the largest number of respondents in this study (59). It is also possible that other forms of bias may have also been an issue. For example, as suicidal behavior is a sensitive topic it is possible that its occurrence was under–reported by respondents (60), although a recent study found that suicidal ideation was reported significantly more often in a web–based survey compared with a computer–assisted telephone interview (CATI), which may have been attributable to not having to disclose sensitive information to an interviewer (61). We also lacked data that would have helped us to better understand the observed associations. For instance, we had no information on stressful life events experienced by the respondents and if any possible stressors were specifically linked to the ongoing pandemic. Similarly, we did not know if any of the respondents had been diagnosed with ADHD and whether they were currently receiving treatment, or whether subjects with ADHD symptoms might have also been suffering from other common mental disorders. This may have been a problem given the large number of subjects with ADHD symptoms who also had depressive and anxiety symptoms. Finally, given the comparatively small number of cases with ADHD symptoms we were not able to stratify the analyses by age or sex which might have also been beneficial given that there is some evidence that the impact of the COVID-19 pandemic may have affected the mental health of the various segments of the Japanese population differently (62).

In conclusion, this study has shown that ADHD symptoms are associated with higher odds for stress and that in turn, stress is an important factor in the association between ADHD symptoms and suicidal ideation. As there is some indication that individuals with ADHD/ADHD symptoms may use maladaptive coping strategies in response to stressful situations (63), some of which may be linked to increased life impairments (64), then the results of this study underscore the importance of identifying sources of stress in this population and of both developing and fostering the use of stress management techniques (65) and adaptive stress coping strategies (64).

## Data availability statement

The raw data supporting the conclusions of this article will be made available by the authors, without undue reservation.

## Ethics statement

The studies involving human participants were reviewed and approved by the Ethics Committee at the National Center of Neurology and Psychiatry, Tokyo, Japan. The patients/participants provided their written informed consent to participate in this study.

## Author contributions

ASt had the study idea, analyzed the data, and wrote the main text. ASh discussed the analysis and commented on the main text for intellectual content. TI, VR, RK, JI, and YI critically reviewed, commented on, and revised the manuscript. TS supervised the project and critically reviewed and revised the manuscript. All authors contributed to the article and approved the submitted version.

## Conflict of interest

The authors declare that the research was conducted in the absence of any commercial or financial relationships that could be construed as a potential conflict of interest.

## Publisher's note

All claims expressed in this article are solely those of the authors and do not necessarily represent those of their affiliated organizations, or those of the publisher, the editors and the reviewers. Any product that may be evaluated in this article, or claim that may be made by its manufacturer, is not guaranteed or endorsed by the publisher.
